# The genetics of ataxia: through the labyrinth of the Minotaur, looking for Ariadne’s thread

**DOI:** 10.1007/s00415-014-7387-7

**Published:** 2014-08-22

**Authors:** M. Mancuso, D. Orsucci, G. Siciliano, U. Bonuccelli

**Affiliations:** Department of Clinical and Experimental Medicine, Neurological Clinic, University of Pisa, Via Roma 67, 56126 Pisa, Italy

**Keywords:** Ataxia, Cerebellum, Diagnosis, Genes, Metabolism

## Abstract

Among the hereditary cerebellar ataxias (CAs), there are at least 36 different forms of autosomal dominant cerebellar ataxia (ADCAs), 20 autosomal recessive cerebellar ataxias (ARCAs), two X-linked ataxias, and several forms of ataxia associated with mitochondrial defects. Despite the steady increase in the number of newly discovered CA genes, patients, especially those with putative ARCAs, cannot yet be genotyped. Moreover, in daily clinical practice, ataxia may present as an isolated cerebellar syndrome or, more often, it is associated with a broad spectrum of neurological manifestations including pyramidal, extrapyramidal, sensory, and cognitive dysfunction. Furthermore, non-neurological symptoms may also coexist. A close integration between clinical records, neurophysiological, neuroradiological and, in some instances, biochemical findings will help physicians in the diagnostic work-up (including selection of the correct genetic tests) and may lead to timely therapy. Some inherited CAs are in fact potentially treatable, and the efficacy of the therapy is directly related to the severity of the cerebellar atrophy and to the time of onset of the disease. Most cases of CA are sporadic, and the diagnostic work-up remains a challenge. Detailed anamnesis and deep investigation of the family pedigree are usually enough to discriminate between acquired and genetic conditions. In the case of ADCA, molecular testing should be guided by taking into account the main associated symptoms. In sporadic cases, a multi-disciplinary approach is needed and should consider the following points: (1) onset and clinical course; (2) associated features; (3) neurophysiological parameters, with special attention to the occurrence of peripheral neuropathy; (4) neuroimaging results; and (5) laboratory findings. A late-onset sporadic ataxia, in which other possible causes have been excluded by following the proposed steps, might be attributable to metabolic disorders, which in some instances may be treatable. In this review, we will guide the reader through the labyrinth of CAs, and we propose a diagnostic flow chart.

## Introduction

The ‘cerebellar ataxias’ (CAs) comprise a wide spectrum of neurological disorders, with ataxia as the main symptom. Ataxia is defined as imbalance and incoordination (e.g., gait ataxia, truncal ataxia) or dysmetria and incoordination of a limb while performing a task (limb ataxia). Gait ataxia is usually secondary to a dysfunction or lesion of the cerebellum or its connections, but patients can also have gait ataxia from peripheral sensory impairment [2]. Neurological examination usually provides an accurate distinction between the two forms [26]. Moreover, CA is typically accompanied by other signs of cerebellar dysfunction, including abnormal eye movements (hypometric or hypermetric saccades, saccadic pursuits), nystagmus of varying types, dysarthria, dysmetria (with kinetic tremor), and dysdiadochokinesis [2].

Once acquired conditions leading to CA are ruled out (see Table 1), making an accurate aetiological diagnosis of ataxia may be a challenge, given the overlap in phenotypes and the various presentations of CAs.

**Table 1:** Acquired ataxias

Type	Subtype(s)
Stroke	–
Toxin-induced	Ethanol
	Gluten (anti-gliadin antibodies)
	Drugs (antiepileptics, lithium salts, antineoplastics, cyclosporine, metronidazole)
	Heavy metals
	Solvents
Immune-mediated	Paraneoplastic syndrome
Infectious/parainfectious diseases	Abscess, cerebellitis
Trauma	–
Neoplastic disorder	Cerebellar tumour, Muir–Torre syndrome
Endocrine	Hypothyroidism
Structural disease	Chiari malformations, agenesis, hypoplasias, dysplasias

Genetic ataxias are frequently chronic and, usually, progressive diseases. Very rarely, genetic ataxias may be episodic (only partially covered in this revision; for more details, see [12]). In apparently sporadic late-onset cases, genetic ataxias can be difficult to discriminate from non-genetic forms, such as multiple system atrophy [20] or progressive supranuclear palsy with predominant CA [17], even though magnetic resonance imaging (MRI) may help physicians to reach the correct diagnosis [21]. Reliable determination of the transmission mode may also require a detailed assessment of the family members [4], and a follow-up period may be needed for an accurate differential diagnosis [44].

A large epidemiological study recently showed that hereditary CAs had a prevalence of 8.9 per 100,000 (5.6 for dominant and 3.3 for recessive ataxias) in a Portuguese population. Machado–Joseph disease (spinocerebellar ataxia type 3; SCA3), Friedreich ataxia and ataxia with oculomotor apraxia were the most frequent forms [3].

Here we focus our attention on the most common genetic forms of CA. Among the presented monogenic causes of ataxia, the diseases that are most frequently encountered in adult neurological practice are Friedreich disease, polyglutamine expansion SCAs and mitochondrial disorders. Table 2 reports the clinical, neuroradiological and biochemical ‘red flags’ for those diseases.

**Table 2:** Some of the possible adjunctive clinical features which can guide molecular diagnosis

Disease	Inheritance	Nervous system	Cardiovascular system	Eye	Biochemical and MRI features	Others
MERRF	Matrilineal	Myoclonus, seizures, hearing loss, myopathy, cognitive impairment, neuropathy	–	–	Lactic acidosis, increased CK	Multiple lipomatosis
MELAS	Matrilineal	Stroke-like seizures, cognitive involvement, hearing loss, neuropathy, migraine, myopathy	Hypertrophic cardiomyopathy, Wolff–Parkinson–White	Eyelid ptosis, ophthalmoparesis, pigmentary retinopathy	Lactic acidosis, cerebral and cerebellar atrophy, white matter lesions, calcifications	Diabetes mellitus, short stature
Leigh syndrome	Matrilineal/recessive	Psychomotor regression, hypotonia, seizures, myoclonus, neuropathy, pyramidal signs	–	Optic atrophy, pigmentary retinopathy	Lactic acidosis, symmetrical lesions in the basal ganglia or brain stem	Early onset
NARP	Matrilineal	Neuropathy	–	Pigmentary retinopathy	–	–
PEO/KSS	Sporadic	Hearing loss, myopathy	Conduction blocks	Eyelid ptosis, ophthalmoparesis, pigmentary retinopathy	–	Short stature
*POLG1*	Recessive (rarely dominant)	Seizures, hearing loss, myopathy, neuropathy	–	Eyelid ptosis, ophthalmoparesis	Cerebral and cerebellar atrophy	–
IOSCA	Recessive	Neuropathy	–	Eyelid ptosis, ophthalmoparesis	Brainstem and cerebellar atrophy	Early onset
*OPA1*	Dominant	Hearing loss, neuropathy	–	Optic atrophy, eyelid ptosis, ophthalmoparesis	–	–
Coenzyme Q10 deficiency	Recessive	Myopathy; pure ataxic forms are frequent	–	–	Low coenzyme Q10 levels in muscle, cerebellar atrophy	Treatable (coenzyme Q10)
Complicated HSPs	Recessive (rarely dominant)	Pyramidal signs, neuropathy	–	–	Thin corpus callosum	–
Friedreich ataxia	Recessive	Pyramidal signs, loss of vibration and proprioceptive sense, areflexia	Hypertrophic cardiomyopathy	–	Brainstem atrophy	Diabetes. Treatable (idebenone)
Vitamin E deficiency	Recessive	Pyramidal signs, loss of vibration and proprioceptive sense, areflexia	–	Pigmentary retinopathy	–	Treatable (vitamin E)
Abetalipoproteinaemia	Recessive	Pyramidal signs, loss of vibration and proprioceptive sense, areflexia	–	Pigmentary retinopathy	Hypocholesterolaemia, acanthocytosis	Malabsorption. Treatable (vitamins)
Refsum disease	Recessive	Neuropathy, hearing loss, anosmia	Cardiomyopathy or arrhythmias	Pigmentary retinopathy	Renal failure	Treatable (diet)
Tay–Sachs disease	Recessive	Areflexia, muscle atrophy, psychiatric involvement, pyramidal signs, seizures	–	–	Cerebellar atrophy	–
Cerebrotendinous xanthomatosis	Recessive	Pyramidal signs, neuropathy, pyramidal signs, seizures, dementia	–	Cataracts	Increased serum cholestanol, cerebral and cerebellar atrophy, white matter lesions	Diarrhoea. Treatable (chenodeoxycholic acid)
NP-C	Recessive	Psychomotor regression, hypotonia, seizures, psychiatric involvement	–	Vertical gaze palsy	–	Splenomegaly. Treatable (miglustat)
Ataxia telangiectasia	Recessive	–	–	Oculomotor apraxia	Increased serum α-fetoprotein	Telangiectasias, immunodeficiency
Ataxia with oculomotor apraxia	Recessive	Neuropathy, extrapyramidal signs, mild cognitive impairment	–	Oculomotor apraxia, nystagmus	Cerebellar vermian atrophy	Treatable? (coenzyme Q10)
ARSACS	Recessive	Pyramidal signs, neuropathy	–	–	Cerebellar vermian atrophy, hyperintensity of the lateral pons	–
ARCA1	Recessive	Dysarthria	–	–	Cerebellar atrophy	–
SCA1	Dominant	Dementia, pyramidal signs, neuropathy	–	Nystagmus, slow saccades	Cerebellar and brainstem atrophy	–
SCA2	Dominant	Dementia, neuropathy, myoclonus	–	Slow saccades	Cerebellar and brainstem atrophy	–
SCA3	Dominant	Parkinsonism, pyramidal signs, neuropathy	–	Nystagmus, diplopia, ophthalmoplegia, eyelid retraction	Cerebellar and brainstem atrophy	–
SCA6	Dominant	–	–	Nystagmus	Cerebellar and brainstem atrophy	–
SCA7	Dominant	Pyramidal signs	–	Retinal degeneration, ophthalmoplegia	Cerebellar and brainstem atrophy	Anticipation
SCA17	Dominant	Dementia, psychosis, chorea, seizures	–	–	Cerebellar and brainstem atrophy	–
DRPLA	Dominant	Dementia, chorea, myoclonus, seizures	–	–	Cerebellar and brainstem atrophy	Anticipation
Episodic ataxia	Dominant	Episodes of vertigo and ataxia	–	–	–	–
FXTAS	X-linked	Tremor, parkinsonism, neuropathy, autonomic dysfunction, dementia	–	–	Hyperintensity in the middle cerebellar peduncles and corpus callosum splenium	–
XLSA/A	X-linked	–	–	–	Anaemia	–

*ARCA1* autosomal recessive cerebellar ataxia type 1, *ARSACS* autosomal recessive spastic ataxia of Charlevoix-Saguenay, *DRPLA* dentate-rubro-pallidoluysian atrophy, *FXTAS* fragile X-associated tremor/ataxia syndrome, *HSP* hereditary spastic paraparesis, *IOSCA* infantile onset spinocerebellar ataxia, *KSS* Kearns–Sayre syndrome, *MELAS* mitochondrial encephalomyopathy, lactic acidosis and stroke-like episodes syndrome, *MERRF* myoclonic epilepsy with ragged red fibres, *NARP* neuropathy, ataxia and pigmentary retinopathy syndrome, *NP-C* Niemann–Pick disease type C, *OPA1* optic atrophy 1 gene-related disease, *PEO* progressive external ophthalmoplegia, *POLG* mitochondrial DNA polymerase gamma related disease (types 1–3, 6, 7, 17), *SCA* spinocerebellar ataxia (types 1–3, 6, 7, 17), *XLSA/A* X-linked sideroblastic anaemia and ataxia

Finally, we also provide a proposed diagnostic flow chart (‘the ball of thread’ of Ariadne) with the intent to help the neurologist Theseus to find his way out of the Minotaur’s ataxic maze.

## Mitochondrial ataxias

The most crucial task of the mitochondrion is the generation of energy as adenosine triphosphate (ATP), by means of the electron transport chain. This metabolic pathway is under the control of both nuclear (nDNA) and mitochondrial (mtDNA) genomes. Mitochondrial diseases are a group of disorders caused by impairment of the mitochondrial respiratory chain. The effects of mutations which affect the respiratory chain may be multisystemic, with involvement of visual and auditory pathways, heart, central nervous system, and skeletal muscle. The estimated prevalence of mitochondrial disorders is 10–20 in 100,000. They are, therefore, one of the most common neuro-genetic conditions.

The genetic classification of mitochondrial disorders distinguishes disorders due to defects in mtDNA from those due to defects in nDNA [25]. The diagnostic process of mitochondrial diseases starts with patient and family history and with physical and neurologic examination. ‘Red flags’ for mitochondrial diseases are short stature, neurosensory hearing loss, ptosis, ophthalmoplegia, axonal neuropathy, diabetes mellitus, hypertrophic cardiomyopathy and migraine. Diagnosis of mitochondrial diseases requires a complex approach including: measurements of serum lactate; electromyography; magnetic resonance spectroscopy; muscle histology and enzymology; and genetic analysis [25]. The treatment of most mitochondrial diseases is still inadequate. In this section we discuss the well-defined forms of mitochondrial disease that most frequently manifest as CA (“Mitochondrial ataxias”) [25].

### mtDNA point mutations

Point mutations in mtDNA are inherited according to the rules of mitochondrial genetics (maternal inheritance, heteroplasmy and the threshold effect, mitotic segregation). Each cell contains multiple copies of mtDNA (polyplasmy), which in normal individuals are identical to one another (homoplasmy). Heteroplasmy refers to the coexistence of two populations of mtDNA––normal and mutated. Mutated mtDNA in a given tissue has to reach a minimum critical number before oxidative metabolism is impaired severely enough to cause dysfunction (threshold effect) [25].

Myoclonic epilepsy with ragged red fibres (MERRF) has a variable onset and is mostly characterized by myoclonus and myopathy (with signs of mitochondrial dysfunction); CA is common [25]. Generalized seizures are another possible adjunctive feature. This syndrome has been associated with various mtDNA point mutations, the most frequent of which are A8344G and T8356C in tRNA lysine. Ito and co-workers [16] reported three MERRF patients (with A8344G mutation) in whom CA was the first symptom; conventional brain MRI showed atrophy of the superior cerebellar peduncles and the cerebellum in all patients, and brainstem atrophy in two patients. A recent large survey of A8344G patients showed that myoclonus was more strictly associated with ataxia than with generalized seizures [24], suggesting that MERRF could be better defined as a myoclonic ataxia rather than a myoclonic epilepsy [23].

Mitochondrial encephalomyopathy, lactic acidosis and stroke-like episodes syndrome (MELAS) indicates a progressive encephalomyopathy characterized by repeated stroke-like events (often involving the posterior cerebral areas), recurrent headache, exercise intolerance, seizures and lactic acidosis. Onset can be before the age of 40. Mutations most frequently associated with this condition are A3243G [22] and T3271C in tRNA leucine (UUR) in the mitochondrial genome. Ataxia may be present, but is not typical [25].

Maternally inherited Leigh syndrome (MILS) has its onset generally during the first year of life. The patient may present with motor retardation, hypotonia, ataxia, epileptic seizures, myoclonus, neuropathy, optic atrophy, pigmentary retinopathy, lactic acidosis and psychomotor regression. It is usually associated with point mutations in the gene *ATP6* (the mtDNA-encoded subunit of complex V of the respiratory chain––the ATP synthase), especially at the T8993G. If the percentage of mutant mtDNA is <90 % (between 70 and 90 %), the clinical picture is more benign, later in onset, and characterized by neuropathy, ataxia and pigmentary retinopathy (NARP syndrome). Marked phenotypic differences even between family members have been reported. *ATP6* mutations have also been associated with cognitive developmental delay, learning disability and progressive ataxia [25].

Other mtDNA point mutations have been linked to complex neurological phenotypes including ataxia, and sequencing of the entire mtDNA (from muscle samples) is indicated in all unusual neurological syndromes, even in the absence of clear maternal inheritance.

### mtDNA sporadic rearrangements

The sporadic occurrence of a mitochondrial disease, such as progressive external ophthalmoplegia (PEO) and Kearns–Sayre syndrome (KSS; ophthalmoplegia associated with pigmentary retinopathy and cardiac conduction block), is suggestive of a single, sporadic, mtDNA deletion.

KSS is associated with a single large-scale mtDNA deletion and is clinically characterized by pigmentary retinopathy and PEO. Associated features are cardiac conduction defects and CA. Onset is often before 20 years of age. Additional features may include growth retardation, short stature, deafness, muscle weakness, endocrinopathies, renal tubular dysfunction and hyperlactacidaemia. A disconnection of Purkinje cells at the dentate nucleus may play a role in the pathogenesis of CA in KSS. CA may also represent a feature of ‘PEO plus’ phenotypes due to single mtDNA deletions [25].

### Polymerase gamma-1 gene-related diseases

Mitochondrial disorders related to nDNA are caused by mutations in structural components or ancillary proteins of the electron transport chain, by defects of the membrane lipid milieu, of coenzyme Q10 biosynthetic genes, and by defects in intergenomic signalling (associated with mtDNA depletion or multiple deletions).

It is now well established that defects in mtDNA replication can lead to mitochondrial dysfunction and disease, including cerebellar dysfunction and ataxia [25]. DNA polymerase gamma (POLG) is the only DNA polymerase in human mitochondria and is essential for mtDNA replication and repair. Nuclear genes encode POLG subunits 1 and 2 (*POLG1* and *POLG2*).

*POLG1* mutations can cause dominant or recessive disorders, frequently associated with severe and multisystem involvement. These forms of mitochondrial disorder are associated with secondary accumulation of multiple deletions in mtDNA. Mutations in the *POLG1* gene have emerged as one of the most common causes of inherited mitochondrial disorders in children and adults. Movement disorders are common [30]. It has recently been observed that *POLG1* mutations are rather common in Central European ataxia patients, causing ataxia with PEO (47 %), psychiatric comorbidities (20 %) and epilepsy (14 %) [36].

The most severe manifestations have been associated with mutations of the ‘spacer’ region of *POLG1* (i.e., ataxia-myopathy syndrome). In a large study, the clinical presentation ranged from the neonatal period to late adult life, with an overlapping phenotypic spectrum from severe encephalopathy and liver failure to late-onset PEO, ataxia, myopathy and isolated muscle pain or epilepsy [14]. A form of chronic *POLG1*-encephalopathy associated with the G1399GA and G2243C mutations is characterized by progressive cerebral and cerebellar atrophy in imaging studies [41].

High carrier frequency of the W748S *POLG1* substitution was reported in control subjects in Finland, explaining the high prevalence of ‘mitochondrial recessive ataxia syndrome’ (MIRAS) in Scandinavia [11]. However, although A467T and W748S are a common cause of ataxia in Scandinavia, they are rare in other European regions. It is, therefore, likely that the high prevalence of MIRAS in Finland and Norway is due to a founder effect.

Another POLG-related recessive ataxia is sensory ataxic neuropathy, dysarthria, and ophthalmoparesis (SANDO), which results from mitochondrial dysfunction due to mtDNA deletions in skeletal muscle. Symptoms present during adulthood [9].

### Infantile onset spinocerebellar ataxia

Infantile onset spinocerebellar ataxia (IOSCA) is a severe autosomal recessively inherited neurodegenerative disorder characterized by progressive atrophy of the cerebellum, brainstem and spinal cord and sensory axonal neuropathy. It is due to recessive mutations in the gene *C10orf2* encoding Twinkle, an mtDNA-specific helicase. Interestingly, different mutations in the same gene cause autosomal dominant progressive external ophthalmoplegia (adPEO) with multiple mtDNA deletions (due to mtDNA replication pausing or stalling) [25].

### Optic atrophy 1 gene-related diseases

A third cause of defects in intergenomic signalling (causing mtDNA multiple deletions) with ataxia arises from optic atrophy 1 (*OPA1*) gene mutations [25]. *OPA1* is the most common cause of autosomal dominant optic atrophy. Extra-ocular neurological complications are common, affecting up to 20 % of all mutational carriers. Bilateral sensorineural deafness beginning in late childhood and early adulthood are the prominent manifestation, followed by a combination of ataxia, myopathy, peripheral neuropathy and PEO from the third decade of life onwards [25].

### Coenzyme Q10 deficiency

Coenzyme Q10 deficiency is a rare autosomal recessive disease which has been associated with five major syndromes: (1) encephalomyopathy (with recurrent myoglobinuria, brain involvement and ragged red fibres); (2) severe infantile multisystemic disease; (3) CA; (4) Leigh syndrome (growth retardation, ataxia and deafness); and (5) isolated myopathy [7]. Primary coenzyme Q10 deficiency due to mutations in ubiquinone biosynthetic genes (i.e. *COQ2*, *PDSS1*, *PDSS2*, *CABC1*) has been identified in patients with the infantile multisystemic and cerebellar ataxic phenotypes. In contrast, secondary coenzyme Q10 deficiency, due to mutations in genes not directly related to ubiquinone biosynthesis (i.e. *APTX*, *ETFDH*, *BRAF*), has been identified in patients with CA, pure myopathy and cardiofaciocutaneous syndrome [25].

Coenzyme Q10 deficiency with CA has also been associated with mutations in the *CABC1*/*COQ8*/*ADCK3* gene [25]. Recently, Gerards and co-workers [10] reported the first nonsense mutations in *CABC1* that most likely led to complete absence of a functional CABC1 protein and suggested that *CABC1* is an important candidate for mutation analysis in progressive CA and atrophy on imaging studies. Patients have a childhood-onset gait ataxia and cerebellar atrophy with slow progression [34]. Horvath and co-workers [13] reported a high frequency of *ADCK3* mutations (4 out of 22 patients) in a cohort of undiagnosed ataxias, highlighting the importance of screening for this potentially treatable form.

Some cases of the ataxic variant of coenzyme Q10 deficiency have been linked to a homozygous mutation in the aprataxin (*APTX*) gene, which causes ataxia oculomotor apraxia type 1 (discussed below). The clinical phenotype of this disorder is homogeneous and is mainly characterized by early-onset cerebellar signs, sensory neuropathy, cognitive decline, and oculomotor deficits. The relationship between aprataxin, involved in DNA repair, and coenzyme Q10 deficiency, if any, is still unclear [25].

CA is the most common phenotype of coenzyme Q10 deficiency, with nearly 100 patients reported to date [7]. Other manifestations include neuropathy, seizures, mental retardation, migraine, psychiatric disorders, muscle weakness and exercise intolerance, congenital hypotonia, upper motor neuron signs, dystonia and chorea, ptosis and ophthalmoplegia, retinitis pigmentosa, optic atrophy, oculomotor apraxia, deafness, lipomatosis, Dandy–Walker syndrome, agenesis of corpus callosum, hypogonadism and other endocrinological problems, hypoalbuminaemia, and hypercholesterolaemia [7]. Initial biochemical evaluation of patients with suspected coenzyme Q10 deficiency should include blood lactate measurement, although normal values do not exclude ubiquinone deficiency. Muscle biopsies occasionally show mitochondrial proliferation or lipid droplets, but can be normal or show only non-specific changes. In patients with the ataxic form, muscle biopsies revealed mitochondrial proliferation, COX-negative fibres, or lipid accumulation in 15/49, and reduced respiratory chain enzyme activities in 27/51 [7]. Direct measurement of coenzyme Q10 in skeletal muscle by high-performance liquid chromatography is the most reliable test for the diagnosis [7]. The diagnosis is reinforced by reduced biochemical activities of respiratory chain complexes, in particular, complexes I + III and II + III. Molecular genetic testing has revealed causative mutations in a small proportion of patients indicating that screening for DNA mutations is not yet effective for diagnosing coenzyme Q10 deficiency, probably because of the large number of proteins involved in ubiquinone biosynthesis and regulation and of secondary coenzyme Q10 deficiencies [7].

Coenzyme Q10 deficiency is a treatable condition, so heightened ‘clinical awareness’ about this diagnosis is essential, especially for paediatricians and infantile neurologists. Varying degrees of coenzyme Q10 deficiency cause variable defects of ATP synthesis and oxidative stress. An early treatment with high-dose coenzyme Q10 may radically change the natural history. Patients with all forms of coenzyme Q10 deficiency have shown clinical improvement with oral coenzyme Q10 supplementation, but cerebral symptoms are only partially ameliorated (probably because of irreversible structural brain damage before treatment and because of poor penetration of coenzyme Q10 across the blood–brain barrier). Patients were given various doses of coenzyme Q10 ranging from 90 to 2,000 mg daily. The small number of patients precluded any statistical analysis but improvement was undoubtedly reported. In several patients coenzyme Q10 supplementation also ameliorated the mitochondrial function (electron transport chain activities, lactic acid values, muscle coenzyme Q10 content). The beneficial effects of exogenous coenzyme Q10 require high doses and long-term administration.

## Hereditary spastic paraparesis with ataxia

Hereditary spastic paraparesis (HSP) describes a heterogeneous group of neuro-genetic disorders caused by degeneration of the corticospinal tracts. The key clinical findings are lower limb spasticity, with hyperreflexia and extensor plantar responses [35]. Urinary urgency is also frequent. Age at onset is extremely variable, from childhood through to late adult life. Traditionally, these conditions have been divided into pure HSPs and complicated HSPs, depending on the presence of adjunctive neurological features such as ataxia, thin corpus callosum, peripheral neuropathy, distal amyotrophy, retinopathy, optic atrophy, extrapyramidal signs, cognitive dysfunction, deafness, and epilepsy [35].

The best-characterized molecular mechanisms in HSPs are impairment of transport of macromolecules and organelles, disturbance of mitochondrial function, or abnormalities of the developing axon [35]. The genetics of HSP is complex and all Mendelian modes of inheritance have been described. Most cases of autosomal dominant HSP are pure, whereas complicated forms tend to be autosomal recessive; *SPG4* (*SPAST*, spastin), *SPG3A* and *SPG31* (*REEP1*) are the most common causes of autosomal dominant pure HSP [35]. Mutations in the *SPG7* gene (paraplegin) are the most common cause of autosomal recessive HSP, with both pure and complicated phenotypes [35], frequently including cerebellar atrophy and cerebellar signs at the neurological examination [32]. Moreover, a significant number of apparently sporadic cases have an *SPG* mutation.

HSP subtypes which more commonly cause a ‘spastic ataxia’ phenotype are the recessive forms *SPG7* and *SPG15* (and the very rare subtypes due to *SPG30* and *SPAX* mutations); these conditions enter the differential diagnosis with Friedreich ataxia and related diseases, autosomal recessive spastic ataxia of Charlevoix-Saguenay (ARSACS), and spinocerebellar ataxia (SCA) subtypes with spasticity (i.e., SCA1, 3, 7, 10, 11, 12) [9].

## Autosomal recessive ataxias

### Friedreich ataxia

Friedreich ataxia, the most common autosomal recessive ataxia in the Caucasian population, is due to mutations in the *FXN* gene, mostly an expanded GAA intronic triplet repeat. Age at onset is 5–25 years. Mixed (cerebellar and sensory) ataxia is the cardinal symptom. The clinical course is variable, but in general 10–15 years after onset patients lose the ability to walk, stand and sit without support. Age at diagnosis, which may incorporate other genetic and environmental factors, may be more important than GAA length in predicting cardiomyopathy, scoliosis, and disease progression [25].

Sensory neurons in the dorsal root ganglia are initially lost, with secondary degeneration of the spinocerebellar and pyramidal tracts, as well as of the dorsal columns. Therefore, Friedreich ataxia is characterized by progressive gait and limb ataxia, dysarthria, loss of vibration and proprioceptive sense, areflexia, abnormal eye movements, and pyramidal signs. Other tracts may be involved (i.e., auditory, optic, etc.). During the disease course, hypertrophic cardiomyopathy may develop. Diabetes, scoliosis, pes cavus, and restless legs syndrome are other possible manifestations [25]. MRI of the brain does not reveal cerebellar atrophy, but mild-to-moderate atrophy of the brain stem and spinal cord.

The genetic abnormality results in the deficiency of frataxin, a protein targeted to the mitochondrion which may represent an activator of oxidative phosphorylation. Although the exact physiological function of frataxin is not known, its involvement in iron–sulfur cluster biogenesis has been suggested. Current evidence suggests that loss of frataxin impairs mitochondrial iron handling and respiratory chain function and contributes to increased oxidative stress and cellular damage. Furthermore, increased mitochondrial iron uptake coupled with decreased utilization and release may lead to mitochondrial iron loading [25].

Treatment options have been mostly directed at antioxidant protection against mitochondrial damage. Idebenone, a coenzyme Q10 derivative, was protective in fibroblasts from patients with Friedreich ataxia and in animal models [25]. Early trials have demonstrated that low-dose idebenone (5 mg/kg per day) could reduce cardiac hypertrophy [25]. A randomized, placebo-controlled trial has been conducted on 48 patients [5]. Treatment with higher doses of idebenone (up to 45 mg/kg) was generally well tolerated and was associated with improvement in neurological function and activities of daily living. The degree of improvement correlated with the dose of idebenone, suggesting that higher doses may be needed to have a beneficial effect on neurological function [5]. Patients with Friedreich ataxia should, therefore, be treated with idebenone because it is well tolerated and may reduce cardiac hypertrophy and, at higher doses (up to 45 mg/kg), it may also improve neurological function.

### Ataxia with vitamin E deficiency

Ataxia with vitamin E deficiency (AVED) is a recessive disorder that presents with a clinically similar phenotype to Friedreich ataxia, with normal cerebellar features at MRI. Decreased visual acuity or retinitis pigmentosa may be an early finding. Cardiomyopathy and diabetes are much less common than in Friedreich ataxia. The disease is caused by mutation of the α-tocopherol transfer protein. Serum concentrations of vitamin E are low. The mechanism underlying this pathogenesis is increased oxidative stress, which might also be a contributory factor in Friedreich ataxia. Supplementation with vitamin E stops progression and can mildly improve CA [8].

### Abetalipoproteinaemia

Abetalipoproteinaemia is an ataxic syndrome caused by mutations in the gene for the large subunit of microsomal triglyceride transfer protein which functions in the assembly of apolipoprotein-B containing very low density lipoproteins and chylomicrons. The neurological phenotype presents before age 20 years, and is similar to Friedreich ataxia, but it is also associated with lipid malabsorption, hypocholesterolaemia, acanthocytosis, and retinitis pigmentosa. Treatment involves dietary modification and vitamin replacement, which may prevent neurological complications if begun early [8].

### Refsum’s disease

Refsum’s disease is a recessive disorder primarily caused by mutation of the gene for the peroxisomal enzyme phytanoyl-CoA hydroxylase (*PHYH*) and is clinically characterized by CA, peripheral polyneuropathy, sensorineural deafness, retinitis pigmentosa and anosmia with skeletal abnormalities, ichthyosis, renal failure, cardiomyopathy or arrhythmias. Onset is typically before age 20 years. Phytanic acid accumulates to high levels in body fat, including myelin. Dietary modifications can stop disease progression [8].

The presence of cerebellar atrophy and/or clinical findings not typically seen in Friedreich ataxia, such as epilepsy or cognitive or psychiatric symptoms [8], should alert physicians to consider the following recessive neurometabolic disorders (discussed below) in the differential diagnosis:

### Late-onset Tay–Sachs disease

Tay–Sachs disease is an autosomal recessive untreatable GM2-gangliosidosis caused by a deficiency of the enzyme β-hexosaminidase A (*HEXA*). It is typically a severe infantile disorder, but the late-onset phenotype presents as either a childhood-onset or adult-onset disease characterized by cerebellar dysfunction, areflexia, proximal muscle weakness with subsequent muscle atrophy and fasciculations, and psychiatric or behavioural problems. Spasticity, seizures, and dementia can also be present in the juvenile-onset form. Cerebellar atrophy on MRI is a typical finding [8].

### Cerebrotendinous xanthomatosis

This disorder is caused by mutation of the mitochondrial enzyme sterol 27-hydroxylase (*CYP27*), part of the hepatic bile acid synthesis pathway, resulting in increases of serum cholestanol and bile alcohols which deposit in CNS tissues. Neurological symptoms generally start around age 20 years and include ataxia with pyramidal or extrapyramidal signs, sensorimotor peripheral neuropathy, seizures, psychiatric problems, and dementia. Associated features include juvenile cataracts, tendon xanthomas, early atherosclerosis, osteoporosis, and chronic diarrhoea. Neuroimaging studies show generalized cerebral and cerebellar atrophy as well as diffuse white matter lesions on MRI. This disease is treatable by bile acid replacement therapy with chenodeoxycholic acid.

### Niemann–Pick disease type C

Niemann–Pick disease refers to a group of autosomal recessive lipid storage disorders associated with a variable degree of neurological manifestations in addition to other organ involvement. Of interest to neurologists is Niemann–Pick disease type C (NP-C) because of the association with neurological manifestations that are not confined to childhood [18]. Neurological symptoms vary with age and include hypotonia, delay in developmental motor milestones, falls, seizures, learning difficulties, ataxia with cognitive deficits, and psychosis [18, 33]. Other typical findings are splenomegaly and vertical supranuclear gaze palsy [27]. Separate articles in this supplement provide detailed descriptions of the clinical and psychiatric symptoms of NP-C (see article by Nia, this issue) and of the genetics and relevant diagnostic procedures associated with this treatable metabolic disorder (see article by Gissen and Mackay, this issue).

### Ataxia telangiectasia

Ataxia telangiectasia results from mutation of a protein kinase involved in the signal transduction cascade triggered by DNA damage (*ATM*). In patients with ataxia telangiectasia, onset of cerebellar dysfunction begins by age 2–3 years and is severely progressive; oculomotor apraxia is common. Associated features include ocular and cutaneous telangiectasias, immunodeficiency, increased risk for leukaemias and lymphomas. High concentrations of serum α-fetoprotein are typically found. No effective treatment is available [8].

### Ataxia with oculomotor apraxia type 1 and 2

A review on ocular movement disorders is available separately in this supplement (see article by Strupp et al. this issue). Ataxia with oculomotor apraxia type 1 presents before the age of 10 years with gait and limb ataxia, sensorimotor neuropathy, eye movement abnormalities including nystagmus, fixation instability, and variable oculomotor apraxia, extrapyramidal signs, and mild cognitive impairment. Cerebellar atrophy (especially of the vermis), hypoalbuminaemia, and hypercholesterolaemia are typical findings. The disease is caused by mutation of the aprataxin gene (*APTX*) which plays a part in DNA repair [8]. In three siblings with mutations in *APTX* gene, coenzyme Q10 supplementation was associated with clear improved ambulation and resolution of seizures in one patient, but further studies are needed [7].

Ataxia with oculomotor apraxia type 2 has a similar phenotype to type 1, but age at onset is in the early teens and laboratory studies show normal albumin and high serum α-fetoprotein concentrations. Ataxia with oculomotor apraxia type 2 could be the second most common autosomal recessive ataxia after Friedreich ataxia in the European population. It is caused by mutations in the gene for senataxin (*SETX*), a DNA/RNA helicase implicated in DNA transcription, DNA repair, and the processing of non-coding RNAs [8].

### ARSACS

ARSACS is due to mutations in the chaperone protein sacsin (*SACS*) and is characterized by progressive cerebellar dysfunction, pyramidal signs and peripheral sensorimotor neuropathy with amyotrophy. Onset is typically at age 1–5 years and MRI shows cerebellar vermian atrophy [8]. A suggestive MRI finding is the hyperintensity of the lateral pons merging into the (thickened) middle cerebellar peduncles [40].

### Autosomal recessive cerebellar ataxia type 1

Autosomal recessive cerebellar ataxia type 1 (ARCA1) is a slowly progressive, adult-onset ataxia with prominent dysarthria and cerebellar atrophy, caused by mutations in the very large *SYNE1* gene [34]. ARCA1 was first reported in patients from the province of Quebec (Canada), but was rarely observed in other populations [29].

A similar form of pure ataxia caused by homozygous *ADCK3* mutations, which has been defined as ARCA2 [34], was discussed above (“Coenzyme Q10 deficiency”).

## Autosomal dominant spinocerebellar ataxias

The designation ‘spinocerebellar ataxias’ (SCAs) indicates the involvement of at least two systems: the spinal cord and the cerebellum [6]. SCAs are progressive neurodegenerative diseases with CA, resulting in unsteady gait, clumsiness, and dysarthria. The cerebellar syndrome may be associated with other neurological signs such as pyramidal or extrapyramidal signs, ophthalmoplegia, and cognitive impairment. Onset is usually during the third or fourth decade of life, but can occur in childhood or old age [6]. Atrophy of the cerebellum and brainstem are most often the prominent features.

Eleven of the 18 known genes are caused by protein polyglutamine expansion [i.e., SCA1, SCA2, SCA3, SCA6, SCA7, SCA17, and dentate-rubro-pallidoluysian atrophy (DRPLA)]. In general terms, polyglutamine-expanded proteins have been reported to increase cellular ROS levels and significantly reduce the mitochondrial electrochemical gradient, inducing DNA damage. The remaining SCAs are caused by non-coding expansions, mutations or large rearrangements in genes with different functions, including mitochondrial activity (SCA28/AFG3L2) [6].

Gait disorders are the initial symptom in two-thirds of patients with polyglutamine expansion SCA [6]. With disease duration, the clinical picture becomes increasingly complex, and this corresponds to the wide distribution of the underlying neuropathology. Hyperreflexia and spasticity are more typical of SCA1, SCA3, and SCA7 [6]. Abnormal eye movements are frequently associated with polyglutamine expansion SCAs. In SCA1, saccade amplitude is increased, resulting in hypermetria and decreased smooth pursuit gain; in SCA2, saccade velocity is substantially decreased and the percentage of errors in antisaccades is high; in SCA3, gaze-evoked nystagmus and hypometric saccades are often present, and smooth pursuit gain decreases greatly; in SCA6, downbeat nystagmus is frequent, and eye movements are similar to those observed in patients with SCA3 [6]. A recent study reported that perverted head-shaking nystagmus could be the most sensitive parameter for SCA6, whereas saccadic intrusions/oscillations were the most sensitive for SCA3; in contrast, a paucity of gaze-evoked nystagmus and dysmetric saccades was more indicative of SCA2 [19].

The presence of associated movement disorders (such as myoclonus, dystonia, chorea, parkinsonism) can help in the diagnostic assessment, considering that they are typical of some forms (e.g. SCA1, 2, 3) [42]. Our group recently reported levetiracetam-responsive myoclonus in a young female patient with SCA15 [31]. Some coexisting movement disorders may be linked to the cerebellar pathology itself (e.g., myoclonus and dystonia), whereas others are more likely related to extracerebellar pathology, and imaging and neuropathological data indeed show involvement of other parts of the motor system (substantia nigra, striatum, pallidum, motor cortex) in some SCA subtypes [42]. When confronted with a patient with an isolated movement disorder without ataxia, there is currently no reason to routinely screen for SCA gene mutations. The only possible exceptions are SCA2 analysis in autosomal dominant parkinsonism (particularly in Asian patients) and SCA17 analysis in the case of a Huntington’s disease-like presentation without an huntingtin mutation [42].

As with age at onset, the clinical picture of polyglutamine expansion SCAs depends on the length of the CAG repeat expansion. Anticipation is most evident in DRPLA and SCA7; paternal expansions are more likely to be unstable during transmission [6].

There are phenotypic differences between polyglutamine expansion SCAs and conventional mutation SCAs. The disease progression is severe and disabling with a life-threatening course in polyglutamine expansion SCAs, in contrast with a slowly progressive course in conventional mutation SCAs, despite early onset [6]. The clinical features of autosomal dominant SCAs are summarized in Table 2.

## X-linked ataxias

### Fragile X-associated tremor/ataxia syndrome

Fragile X-associated tremor/ataxia syndrome (FXTAS) is a late-onset neurodegenerative disorder that affects individuals who are carriers of premutation expansions (55–200 CGG repeats) in the 5′ untranslated region of the *FMR1* (fragile X mental retardation 1) gene. This disease is characterized by a late-onset intention tremor and gait ataxia, with possible parkinsonism, neuropathy, autonomic dysfunction, and dementia. Penetrance is incomplete and higher in males. It is one of the most common hereditary late-onset CAs, with a frequency of about 1 in 3,000 [34]. Symmetric regions of increased T2 signal intensity in the middle cerebellar peduncles and adjacent cerebellar white matter are considered typical of this disease [9]. A recent survey reported that more than 40 % of patients had no family history of fragile X syndrome and more than 80 % had tremor and/or peripheral neuropathy; 60 % of patients had parkinsonism; corpus callosum splenium hyperintensity was as frequent as middle cerebellar peduncle hyperintensity (about 65 %) [1].

### X-linked sideroblastic anaemia and ataxia

X-linked sideroblastic anaemia and ataxia (XLSA/A) is a recessive disorder characterized by an infantile to early childhood onset of non-progressive CA and mild anaemia with hypochromia and microcytosis. A gene encoding an ATP-binding cassette (*ABC7*) transporter, mapped to Xq13, has been linked to this disease. ABC7 localizes to the mitochondrial inner membrane and is involved in iron homeostasis [25].

## Episodic ataxias

Episodic ataxias are characterized by recurrent, discrete episodes of vertigo, ataxia, and often migraine headaches and nausea. The ataxia may or may not be progressive. Episodic ataxia genes code for ion channels or glutamate transporters [34]. Episodic ataxia type 1 is clinically defined by brief episodes of ataxia where the patient is unaffected in between; it is due to *KCNA1* gene mutations. In episodic ataxia type 2 the attacks can last longer and there is usually a background ataxia on examination; the causal gene is *CACNA1A*. In the interictal interval, myokymia is present in hand muscles when analysed by EMG in the type 1 form, whereas type 2 patients present with interictal gaze-evoked nystagmus, downbeat nystagmus, and other cerebellar ocular motor disorders [9]. There are a few other forms that are extremely rare. The first-line treatment nowadays is 4-aminopyridine; second-line therapies include acetazolamide and diclofenamide [15, 37, 38].

## How to treat ataxia

Apart from the availability of specific treatment options for some of the diseases discussed above, treatment of degenerative CA remains very difficult [15]. To date, no medication has been proven effective. Aminopyridines and acetazolamide, which are beneficial in patients with episodic ataxia type 2, are the only exception [15]. Aminopyridines are also effective in a subset of patients presenting with downbeat nystagmus [15]. Acetyl-dl-leucine could improve ataxic symptoms in some patients, but controlled trials are still needed [39]. The mainstays of treatment for degenerative CA are currently physiotherapy, occupational therapy, and speech therapy, but evidence-based guidelines for the physiotherapy of degenerative CA need to be developed [15].

## How to approach a patient with ataxia

Although the majority of disease genes have been found in the past two decades, over the past 3 years the genetics has undergone a methodological revolution [34]. New DNA sequencing technologies allow the study of large proportions of the genome in a rapid and affordable way. Even if a specific challenge of next-generation sequencing data is pathogenicity interpretation, a recent study showed that genetic testing using targeted capture followed by next-generation sequencing was efficient, cost-effective, and enabled a molecular diagnosis in many cases with undiagnosed ataxia [28]. Exome and targeted sequencing has recently identified a few new genes causing ataxia (e.g. *TGM6*, *ANO10*, *SYT14*, and rundataxin) [34]. This approach is likely to continue to discover new ataxia genes and make screening of causative genes more effective in terms of cost and time to diagnosis.

However, most cases of CA are sporadic, and the diagnostic work-up to date remains a challenge, with at least 50 % of patients remaining without a molecular diagnosis.

**Fig. 1:**
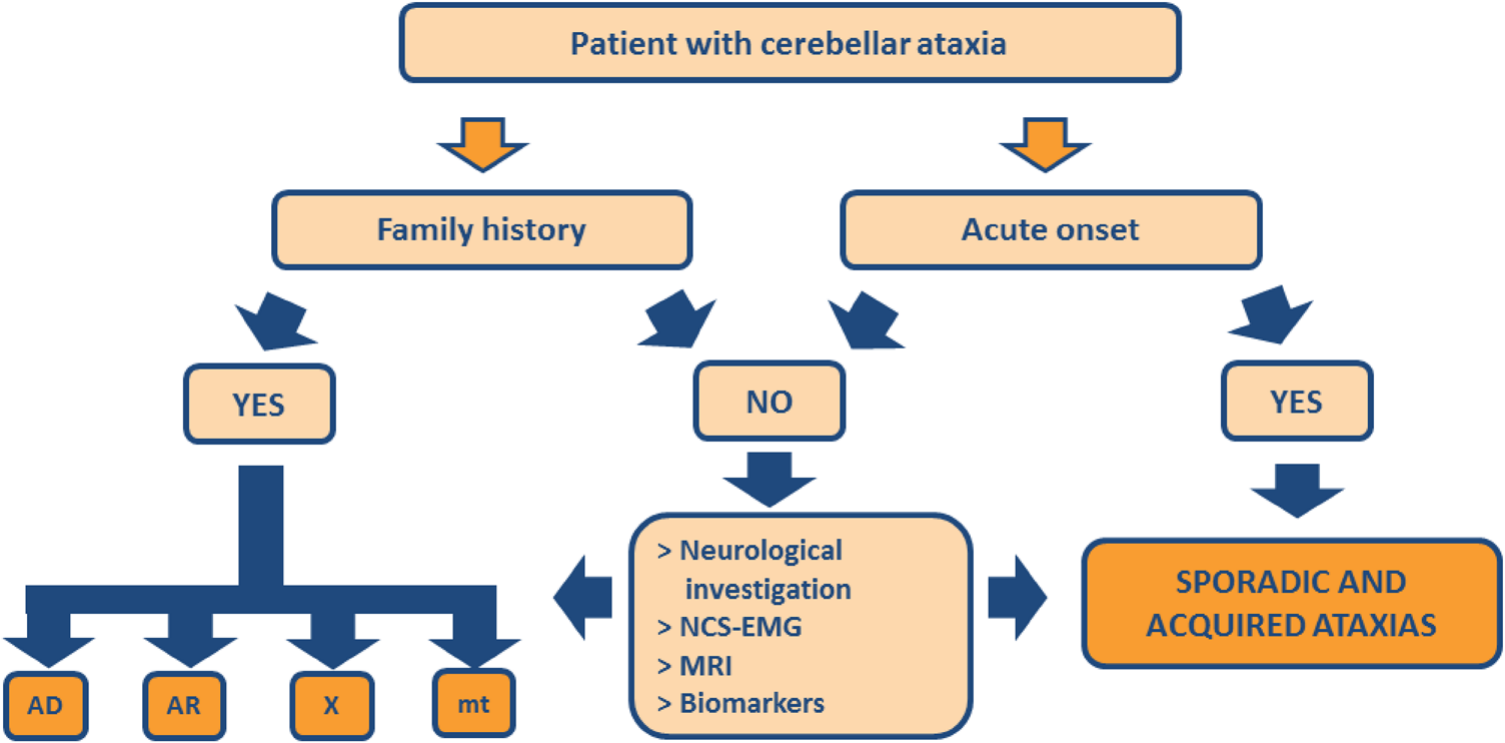
Discrimination of acquired and hereditary conditions. *AD* autosomal dominant, *AR* autosomal recessive, *MRI* magnetic resonance imaging, *mt* mitochondrial, *NCS–EMG* nerve conduction studies and electromyography, *X* X-linked

Detailed anamnesis and deep investigation of the family pedigree are usually enough to discriminate between acquired and genetic conditions (Fig. 1; Table 1). Once the acquired conditions have been ruled out, and the suspicion of an inherited condition raised, the pedigree analysis might already be indicative of the pattern of inheritance.

**Fig. 2:**
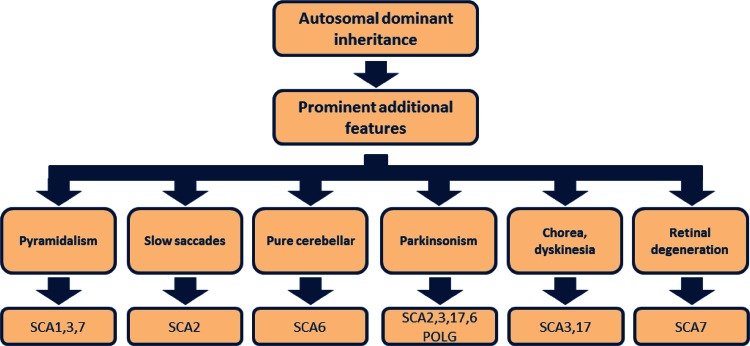
Differential neurological signs in autosomal dominant cerebellar ataxia. *SCA* spinocerebellar ataxia (types 1–3, 6, 7, 17), *POLG* mitochondrial DNA polymerase gamma

In the case of autosomal dominant CA, molecular testing should be guided by taking into account the main associated symptoms (Fig. 2) [9].

**Fig. 3:**
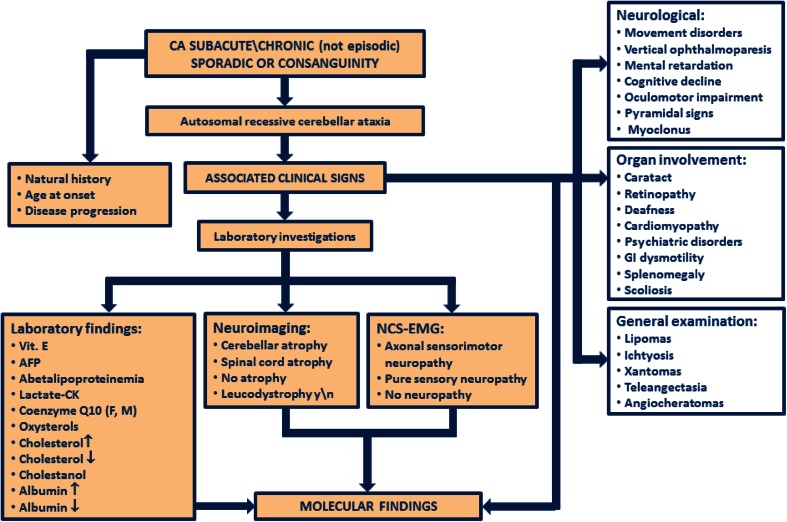
Multidisciplinary approach to differential diagnosis of cerebellar ataxia. *AFP* alpha feta protein, *CA* cerebellar ataxia, *F* fibroblasts, *M* skeletal muscle, *NCS–EMG* nerve conduction studies and electromyography

In sporadic cases, we propose here a multidisciplinary approach (Fig. 3), where the neurologist should always deal at least with the following points to get out of the ataxic maze: (1) onset and clinical course of the disease; (2) associated clinical features (not only neurological, but also psychiatric, ophthalmological, skin observation, etc.); (3) neurophysiological parameters, with special attention to the nerve conduction studies and the occurrence of peripheral neuropathy (sensory or sensory motor neuropathy) or not; (4) neuroimaging results (presence or absence of cerebellar atrophy and leukodystrophy) [43]; (5) laboratory findings. Regarding the laboratory tests, even though not all of the presented tests are available in routine laboratories, detailed research of at least some of those parameters may lead the physician to a correct diagnosis. We should all be aware that a late-onset sporadic ataxia, in which other possible causes have been excluded by following the proposed steps, might be attributable to a metabolic disorder (e.g. NP-C or mitochondrial diseases), which in some instances may be treatable.
